# A computational analysis of the glycoprotein lipoprotein receptor-related protein 1 structure and the role of glycans as quaternary glue

**DOI:** 10.1093/bioinformatics/btag357

**Published:** 2026-06-04

**Authors:** Gian Marco Tuveri, Marco Basile, Silvia Acosta Gutiérrez, Marius Kausas, Silvia Pujals, Xiaohe Tian, Giancarlo Franzese, Lorena Ruiz Pérez, Giuseppe Battaglia

**Affiliations:** Institute for Bioengineering of Catalunya (IBEC), The Barcelona Institute of Science and Technology (BIST), Barcelona 08028, Spain; Institute of Nanoscience and Nanotechnology (IN2UB), University of Barcelona, Barcelona 08028, Spain; Department of Condensed Matter Physics, University of Barcelona, Barcelona 08028, Spain; Institute for Bioengineering of Catalunya (IBEC), The Barcelona Institute of Science and Technology (BIST), Barcelona 08028, Spain; Institute of Nanoscience and Nanotechnology (IN2UB), University of Barcelona, Barcelona 08028, Spain; Department of Biomedicine, University of Barcelona, Barcelona 08028, Spain; Institute for Bioengineering of Catalunya (IBEC), The Barcelona Institute of Science and Technology (BIST), Barcelona 08028, Spain; InstaDeep Ltd, London NW1 3BF, United Kingdom; Institute for Bioengineering of Catalunya (IBEC), The Barcelona Institute of Science and Technology (BIST), Barcelona 08028, Spain; Vianautis Bio Ltd, Cambridge CB22 3FT, United Kingdom; Department of Biological Chemistry, Institute for Advanced Chemistry of Catalonia, Barcelona 08034, Spain; Department of Radiology and Huaxi MR Research Center (HMRRC), Functional and Molecular Imaging Key Laboratory of Sichuan Province, West China Hospital of Sichuan University, Chengdu, Sichuan Province 610000, China; Department of Radiology and National Clinical Research Center for Geriatrics, Frontiers Science Center for Disease-Related Molecular Network, State Key Laboratory of Biotherapy, West China Hospital of Sichuan University, Chengdu, Sichuan Province 610000, China; Institute of Nanoscience and Nanotechnology (IN2UB), University of Barcelona, Barcelona 08028, Spain; Department of Condensed Matter Physics, University of Barcelona, Barcelona 08028, Spain; Institute for Bioengineering of Catalunya (IBEC), The Barcelona Institute of Science and Technology (BIST), Barcelona 08028, Spain; Department of Applied Physics, University of Barcelona, Barcelona 08028, Spain; Institute for Bioengineering of Catalunya (IBEC), The Barcelona Institute of Science and Technology (BIST), Barcelona 08028, Spain; Catalan Institution for Research and Advanced Studies (ICREA), Barcelona 08010, Spain

## Abstract

**Motivation:**

The low-density lipoprotein receptor-related protein 1 (LRP1) plays a critical role in development and transport across the blood–brain barrier (BBB), yet its molecular architecture has remained unresolved due to the absence of an experimentally determined structure.

**Results:**

Using homology modeling and neural network-based structure prediction algorithms, complemented with molecular dynamics (MD) simulations, we propose atomistic models of both monomeric and dimeric LRP1 forms. The simulations reveal a plausible dimerization mechanism and provide insight into the dynamic behavior of its flexible domains under physiological conditions. We estimated the energy required to disrupt the non-covalent interactions linking LRP1’s α and β chains to be 180±2 k_B_ T. MD simulations further highlight the fundamental role of glycans in stabilizing the dimeric quaternary structure by increasing intra-dimer contacts. The resulting structural models also provide experimentally testable estimates of LRP1 size, domain organization, and interface stability that may guide future imaging and mutagenesis studies. This study enhances our molecular understanding of LRP1-mediated transport across the BBB and the role of glycosylation in protein–protein interactions, opening new avenues for targeted drug design strategies.

**Availability and implementation:**

The monomeric and dimeric LRP1 models are available in ModelArchive under the accession codes ma-k8036 and ma-ubwf7, respectively.

## 1 Introduction

The low-density lipoprotein receptor-related protein 1 (LRP1) is a multifunctional endocytic receptor involved in diverse physiological processes, including cellular signaling, lipid metabolism, and the clearance of extracellular proteins. It has been implicated in a wide range of pathologies, such as cancer, atherosclerosis, and neurodegenerative diseases (Gonias and Campana 2014, Emonard and Marbaix 2015, Lillis *et al.* 2015, Au *et al.* 2017, Shinohara *et al.* 2017, Potere *et al.* 2019). LRP1 is widely expressed in mammalian tissues, and its genetic deletion is embryonically lethal due to impaired vascular and neural development (Liu *et al.* 2010, Nakajima *et al.* 2014). As a result, LRP1 is recognized as a critical regulator of cellular homeostasis and signaling pathways (Lillis *et al.* 2008, Gonias and Campana 2014, Potere *et al.* 2019). The receptor can interact with more than forty distinct ligands (Lillis *et al.* 2008, Gonias and Campana 2014, Potere *et al.* 2019), a remarkable binding promiscuity likely enabled by its large size (4544 amino acids) and modular domain organization.

**Figure 1:**
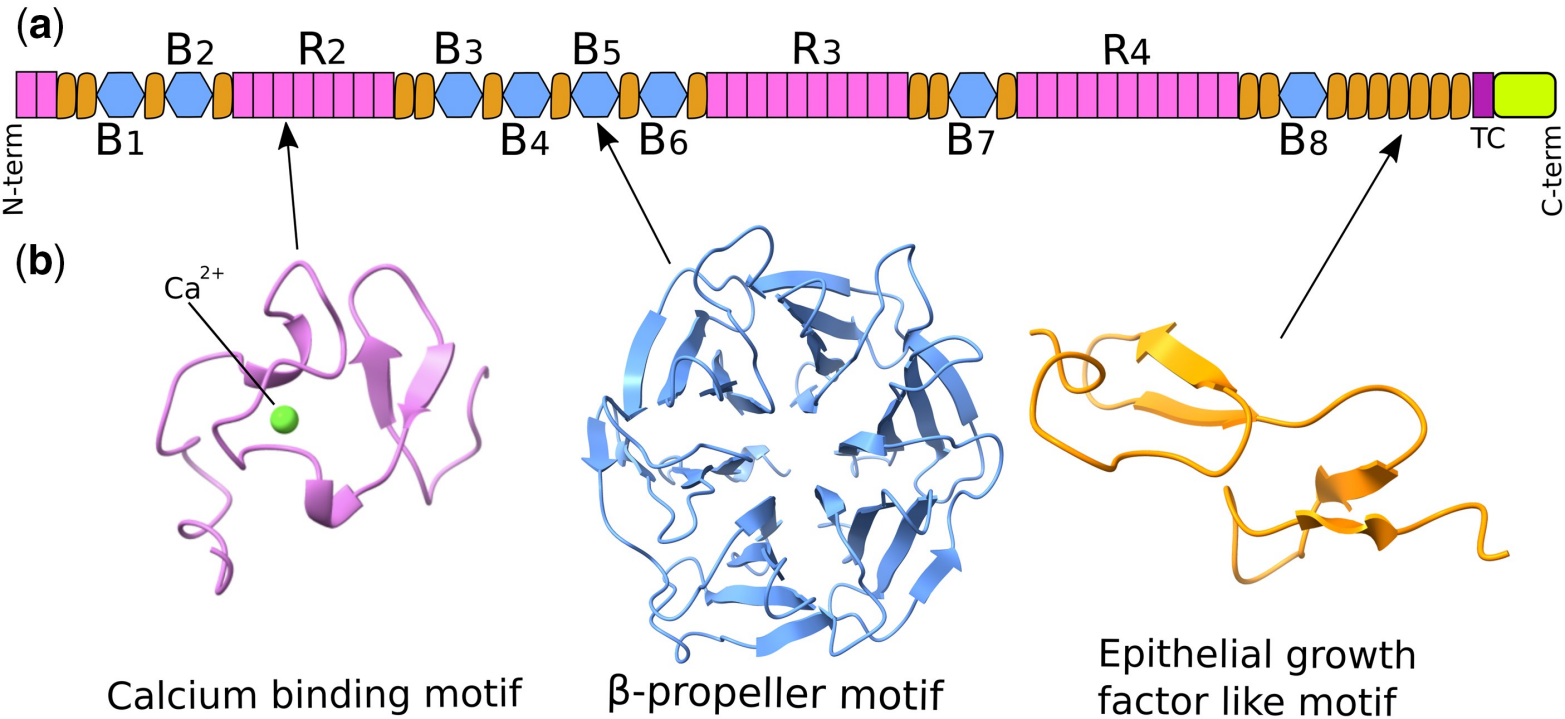
(a) The colored scheme shows the motif sequence in LRP1. Each color corresponds to a different structural motif: from right to left the calcium-binding, β-propeller, and EGF-like domains found in the LRP family. (b) The three structural motifs observed in another member of the LDL receptor family: LRP2 (8EM4).

LRP1 is synthesized as a single-chain precursor that is cleaved by furin in the trans-Golgi compartment into two non-covalently associated subunits: a 515 kDa extracellular α-chain and an 85 kDa transmembrane β-chain (Willnow *et al.* 1996, Lillis *et al.* 2008, Gonias and Campana 2014). The extracellular portion, or ectodomain, was visualized by Nardis *et al.* (2017) using negative-stain electron microscopy and small-angle X-ray scattering (SAXS), revealing an extended, flexible monomeric structure of approximately 35 nm. Each subunit contains three conserved motif types characteristic of the LDL receptor family (Fig. 1): (i) calcium-binding (CB) motifs featuring six conserved cysteines forming three disulfide bridges and acidic residues that coordinate Ca^2+^ ions (Simonovic *et al.* 2001, Rudenko *et al.* 2002); (ii) β-propeller motifs composed of six β-sheets arranged in a radial fold (Springer 1998); and (iii) epidermal growth factor (EGF)-like domains, also stabilized by disulfide bridges but lacking metal-binding sites (Jeon *et al.* 2001).

Although the architecture of these local motifs is well understood, the overall tertiary and quaternary organization of full-length LRP1 remains unresolved. Recently, cryo-electron microscopy studies of the closely related receptor LRP2 (megalin; PDB ID: 8EM4) revealed a homodimeric assembly stabilized by hydrogen bonds and salt bridges (Passarella *et al.* 2022, Beenken *et al.* 2023). LRP2 shares extensive sequence and structural similarity with LRP1 and binds an overlapping set of ligands (Spuch *et al.* 2012). These findings raise the possibility that LRP1 could also form higher-order structures. Such a dimeric organization would have important functional implications, as LRP1 is known to cluster on the plasma membrane and engage in multivalent interactions with large or repetitive ligands.

Both LRP1 and LRP2 are heavily glycosylated receptors. Covalently attached N-glycans are known to modulate protein folding, inter-domain interactions, and receptor stability by shielding proteolytic sites and stabilizing specific conformations (Varki 2017). In the context of LRP1, glycosylation could influence dimer formation or maintain receptor conformation under physiological conditions, but its structural role remains unexplored. More broadly, glycan-mediated stabilization mechanisms have been observed in several viral and membrane proteins, including the SARS-CoV-2 spike, where glycans regulate folding, receptor binding, and immune evasion (Acharya *et al.* 2021, Acosta-Gutiérrez *et al.* 2022, Arora *et al.* 2024). These examples emphasize the need to include glycosylation explicitly when modeling large extracellular receptors such as LRP1.

Beyond its structural complexity, LRP1 plays a fundamental role in receptor-mediated transport across the blood–brain barrier (BBB), contributing to the clearance of amyloid β and tau, two major pathological proteins in Alzheimer’s disease (Chen *et al.* 2025). In previous work, we demonstrated that LRP1 participates in the formation of tubular transcytotic vesicles stabilized by the F-BAR protein syndapin-2, enabling efficient trafficking of misfolded proteins and nanoparticles across the BBB (Tian *et al.* 2020). These transport processes depend on multivalent interactions, where multiple LRP1 molecules engage simultaneously with one ligand or nanoparticle, and their strength is governed by the receptor’s spatial organization.

In this study, we investigate the molecular organization of LRP1 through a combination of super-resolution microscopy, structure prediction, and molecular dynamics (MD) simulations. We present atomistic models of both monomeric and dimeric LRP1 forms, the latter derived by homology modeling based on LRP2. Through extensive MD analysis, we examine the dynamics of flexible domains, calcium coordination, and glycan-mediated stabilization, providing a structural framework for understanding how LRP1’s architecture supports its receptor-mediated transport functions at the BBB.

## 2 Materials and methods

### 2.1 Model construction

The monomeric structure of LRP1 was generated based on its sequence and the known organization of LDL receptor family motifs (Herz *et al.* 1988). Individual domains were predicted using RoseTTAFold (Baek *et al.* 2021) and AlphaFold2 (Jumper *et al.* 2021), then assembled in Chimera (Pettersen *et al.* 2004) following motif order.

**Figure 2:**
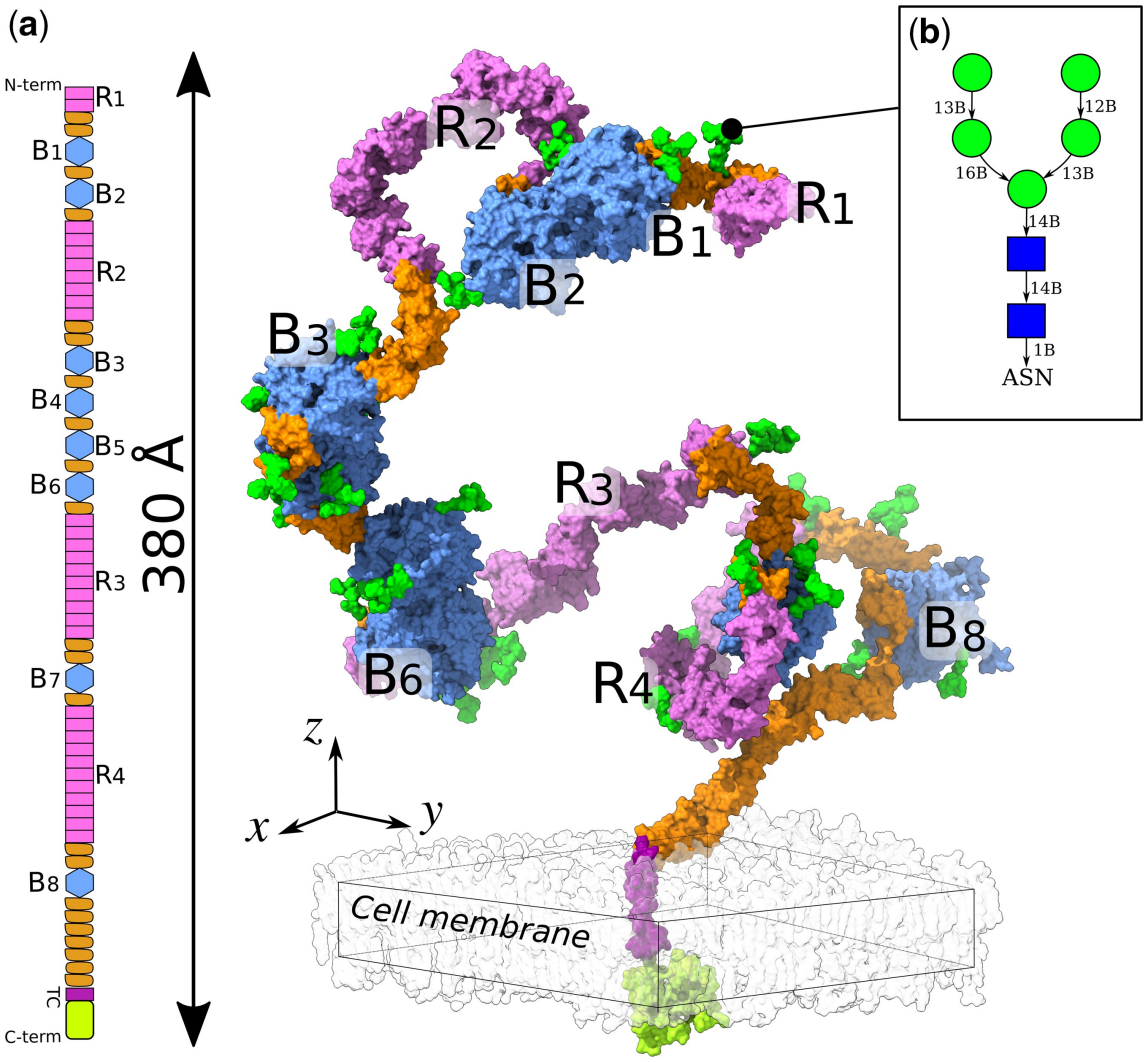
(a) The proposed structure of the LRP1 monomer inserted in the cell membrane is represented by a vdW surface. Each local domain follows the colors in (a). Glycans are represented in light green surface. The membrane is shown schematically to indicate receptor orientation and was not included explicitly in MD simulations. (b) 2D representation of the GlcNAc(2)Man(5) glycans used in this work.

The global model quality score reported by QMEANDisCo (Studer *et al.* 2020) is 0.54 ± 0.05. Calcium ions were positioned at conserved coordination sites identified by sequence alignment (Simonovic *et al.* 2001, Beenken *et al.* 2023), and disulfide bridges were added to preserve domain connectivity. Along the protein, 52 predicted N-linked glycans (LRP1 Uniprot code Q07954) were attached using CHARMM-GUI (Jo *et al.* 2008), employing simplified high-mannose GlcNAc_2_Man_5_ structures (Fig. 2b); the list of glycosylation sites is shown in Table 2, available as supplementary data at *Bioinformatics* online. High-mannose glycans were selected as a simplified and compositionally consistent model because they represent the common structural core shared by most complex N-glycoforms and are well parameterized for MD simulations. During model construction, minor adjustments were applied to remove steric clashes and ensure physically consistent connectivity between domains, while preserving the predicted secondary and tertiary structures of individual motifs. These adjustments were subsequently relaxed through MD simulations. This approach differs from manual rearrangement of full-length predictions, as it does not impose arbitrary inter-domain orientations but instead allows the system to evolve toward energetically favorable conformations. This domain-wise assembly followed by MD relaxation is consistent with previous approaches used for extended receptors, where global structure prediction is limited by inter-domain flexibility (Tavanti *et al.* 2025).

The dimeric LRP1 model was obtained by homology modeling from the mouse LRP2 template (PDB 8EM4) using MODELLER (Webb and Sali 2016), with missing segments predicted by RoseTTAFold. The global model quality score reported by QMEANDisCo (Studer *et al.* 2020) is 0.48** ± **0.05. The same transmembrane and intracellular domains predicted with RoseTTAFold were consistently used in both the monomeric and dimeric models. A detailed structural and functional characterization of these regions is beyond the scope of the present work, which focuses on the extracellular organization and ligand-binding properties of LRP1.

Both models were energy-minimized in implicit solvent using OpenMM (Eastman *et al.* 2017) with the AMBER ff14SB (Maier *et al.* 2015) and GLYCAM06j-1 (Kirschner *et al.* 2008) force fields (see SI for details).

### 2.2 MD simulations

MD simulations were performed for structural refinement rather than exhaustive sampling of global conformational dynamics. Quantitative analyses were therefore performed on selected subdomains, enabling adequate sampling while preserving local structural accuracy. All MD simulations were performed using GROMACS 2021–2022 (Abraham *et al.* 2015) and the CHARMM36m force field (Huang *et al.* 2017). Systems were solvated with TIP3P water (MacKerell *et al.* 1998) and 0.15 M KCl, neutralized, and equilibrated under standard conditions (303 K, 1 bar). Production trajectories were run for 100–500 ns depending on system size. Details of equilibration protocols, thermostats, and barostats are provided in the Supporting Information.

#### 2.2.1 Chain separation energy

To quantify the stability of the non-covalent interactions between LRP1’s α and β chains, we performed umbrella sampling on the isolated B8 domain (Asp3779–Gln4235). Configurations were extracted along a 5 nm reaction coordinate, and the potential of mean force (PMF) was obtained using the Weighted Histogram Analysis Method (WHAM) (Kumar *et al.* 1992). Uncertainties were estimated by Bayesian bootstrap analysis (Hub *et al.* 2010).

#### 2.2.2 Domain flexibility

To examine LRP1 flexibility, three regions (R2, R3, and R4) containing consecutive CB and EGF-like motifs were simulated in isolation. Each system was initialized in an extended conformation and equilibrated for 100 ns. End-to-end distances (d_EE_) and root mean square fluctuations (RMSF) were computed using MDAnalysis (Michaud-Agrawal *et al.* 2011) and VMD (Humphrey *et al.* 1996).

#### 2.2.3 Calcium stability

The stability of the calcium coordination cage was assessed for CB7 using umbrella sampling with WHAM (Kumar *et al.* 1992). The PMF between bound and unbound states was determined, and ion–cage interactions were characterized by the positions of coordinating acidic residues.

#### 2.2.4 Dimeric canopy simulations

The canopy region of the dimer (residues 1140–2522 of both monomers) was simulated in four glycosylation states: fully glycosylated, asymmetric (two cases), and non-glycosylated. Glycans (highlighted in red in Table 2, available as supplementary data at *Bioinformatics* online) were added with Glycosylator (Lemmin and Soto 2019) for major control over the sterics and to avoid atomic clashes. Glycan anchored to Asn1155 was ignored for steric reasons (multiple clashes with adjacent Asn1154); however, this glycan is not located at the interface of β-propellers. Systems were built using CHARMM-GUI and equilibrated under identical conditions. Production runs extended up to 700 ns. Inter-protomer distances, RMSD, and hydrogen-bond statistics were analyzed with MDAnalysis (Michaud-Agrawal *et al.* 2011).

### 2.3 Evolutionary conservation analysis

Evolutionary conservation was evaluated using ConSurf (Glaser *et al.* 2003), based on a multiple sequence alignment of LRP1 orthologues retrieved from UniProt. Residue conservation scores were mapped onto the canopy β-propellers to identify conserved structural and interfacial motifs. Complete methodological details and input files are provided in the Supporting Information.

## 3 Results

### 3.1 LRP1 clusters on the surface of brain endothelial cells

**Figure 3:**
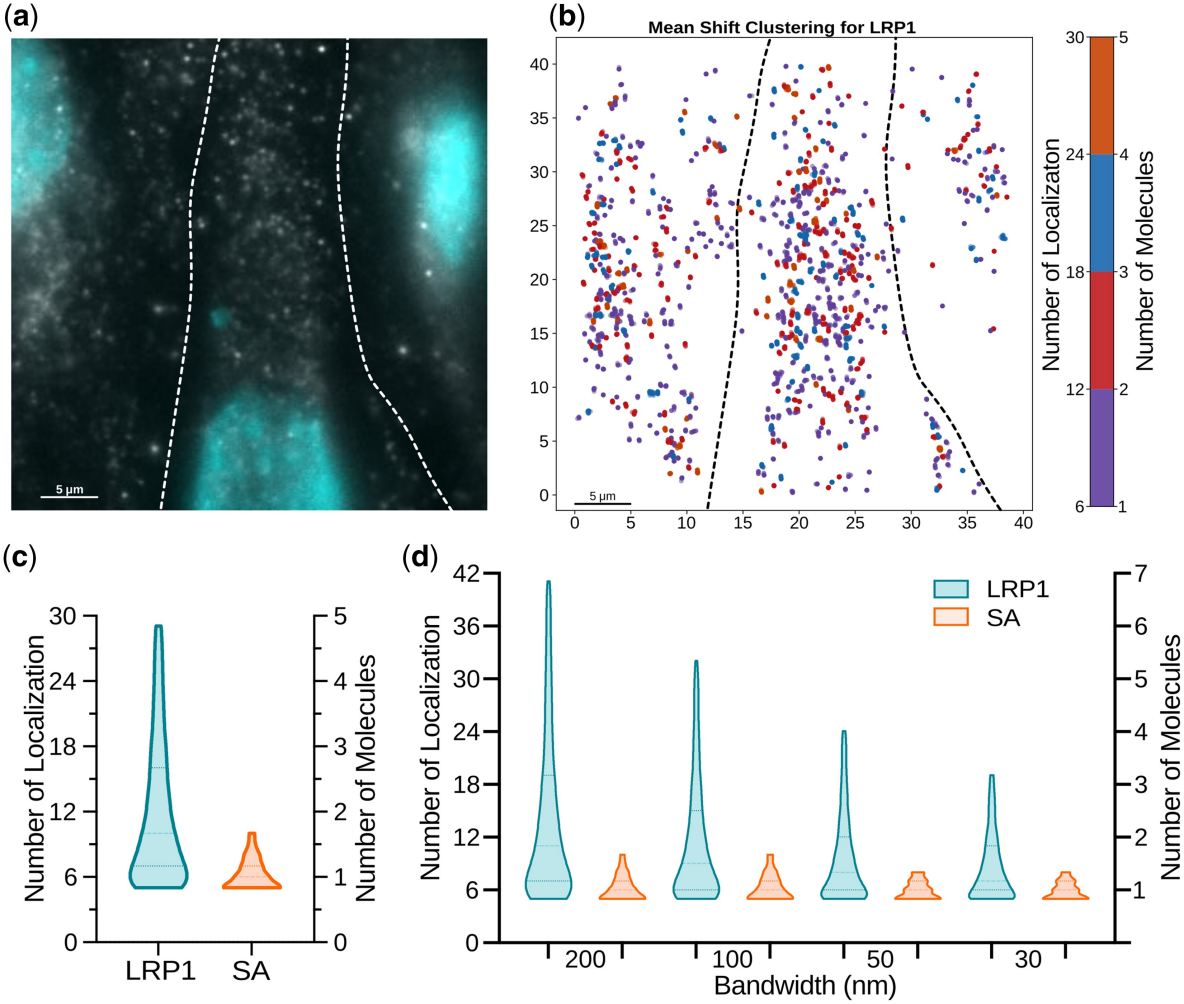
(a) Low-resolution fluorescent image taken before photobleaching the region of interest (ROI) where nuclei , LRP1 , and cell borders are highlighted, (b) STORM image and Mean Shift Clustering analysis of the same ROI, with bandwidth at 200 nm (c) Violin plot comparison of the clustered number of localization (NOL) and number of molecules (NOM) for LRP1 and single secondary antibody (SA) of (b, d) NOL and NOM clusters distributions of LRP1 and SA at different clustering bandwidths of five different images.

Using stochastic optical reconstruction microscopy (STORM), we investigated the spatial organization of LRP1 on brain endothelial (bEnd3) cells with nanometer precision (Fig. 3a). Cells were labelled with an Alexa-647–conjugated secondary antibody, and the system was first calibrated by imaging the antibody at highly diluted concentrations, as described in Section 2. This calibration, supported by Mean Shift Clustering analysis, established that a single antibody produced a median of six fluorescence localizations, providing a reference to distinguish single molecules from clusters (Fig. 3c).

When imaging LRP1 on the cell surface, we observed discrete clusters with up to 30 fluorescence localizations (Fig. 3b). Varying the clustering bandwidth between 30 and 200 nm confirmed that, even when signals merged at larger bandwidths, LRP1 maintained a non-uniform distribution distinct from that of isolated antibodies (Fig. 3d). These findings suggest that LRP1 forms higher-order assemblies, with multiple receptors colocalizing into small, discrete surface clusters, potentially including dimeric or multimeric arrangements.

### 3.2 Atomistic model of monomeric LRP1

To construct an atomistic model of LRP1, we evaluated the performance of AlphaFold2 (Jumper *et al.* 2021) and RoseTTAFold (Baek *et al.* 2021) in predicting local tertiary motifs (Fig. 1b). Both algorithms reproduced known CB, β-propeller, and EGF-like motifs with high confidence and close agreement (Figs 1 and 2, available as supplementary data at *Bioinformatics* online), consistent with available structures from the homologous receptor LRP2 (8EM4) (Beenken *et al.* 2023). Full-length AlphaFold3 predictions of LRP1 exhibited structural inconsistencies, including steric clashes and unrealistic domain packing (Fig. 3, available as supplementary data at *Bioinformatics* online), preventing their direct use.

**Figure 4:**
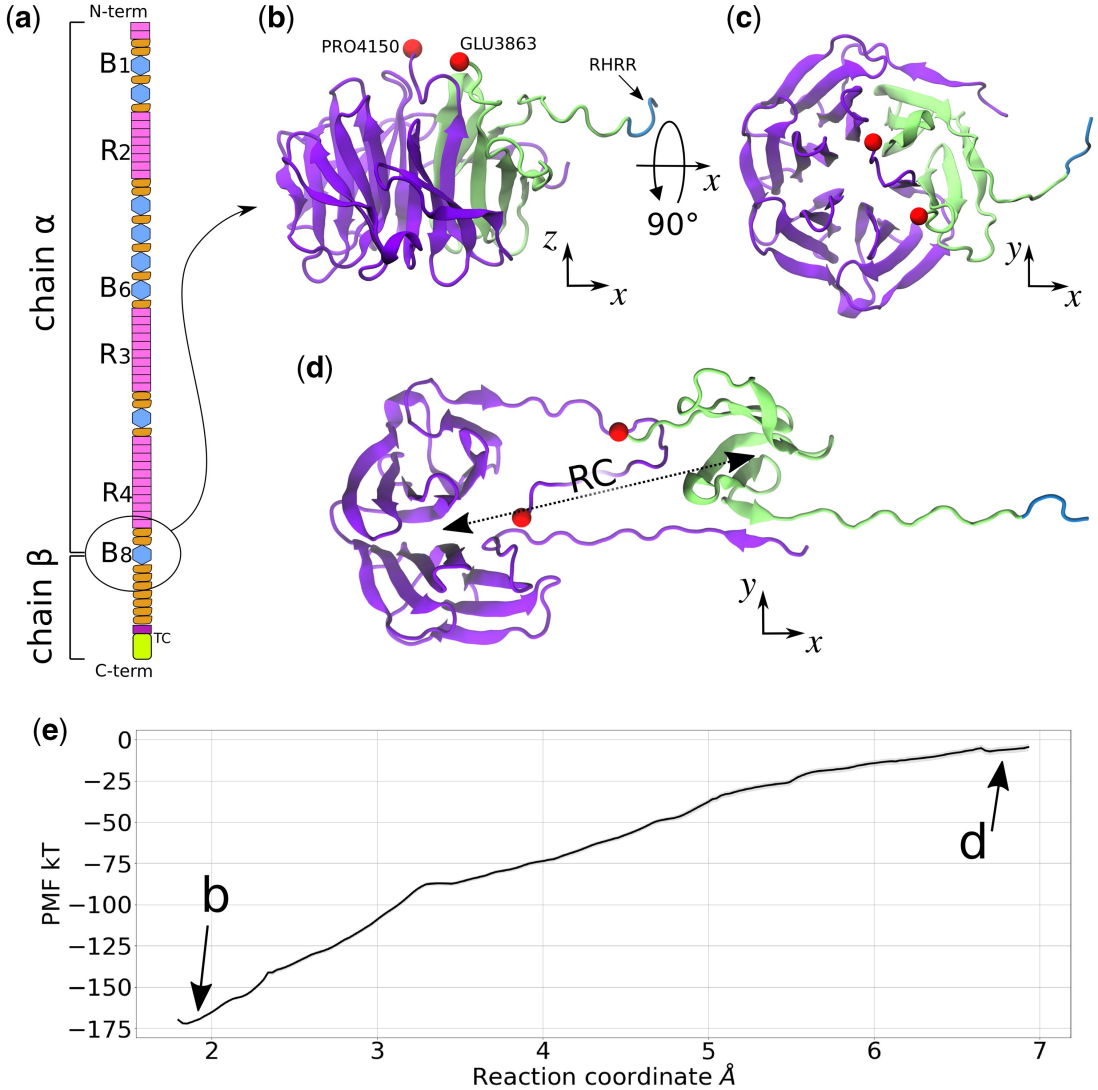
(a) The scheme shows the location, along the LRP1 motif sequence, of β-propeller 8 (B8). In correspondence of this motif, the protein is divided into α and β chains. (b, c) B8 structure prediction from AlphaFold2, depicted as a cartoon in lime (α chain) and violet (β-chain) with side (b) and top (c) perspective. The RHRR sequence is depicted in blue; the extreme amino acids of the unit are depicted in red spheres. d) B8 at the extreme distance of α and β centers of mass. e) The potential of mean force for the α and β centers of mass distance.

**Figure 5:**
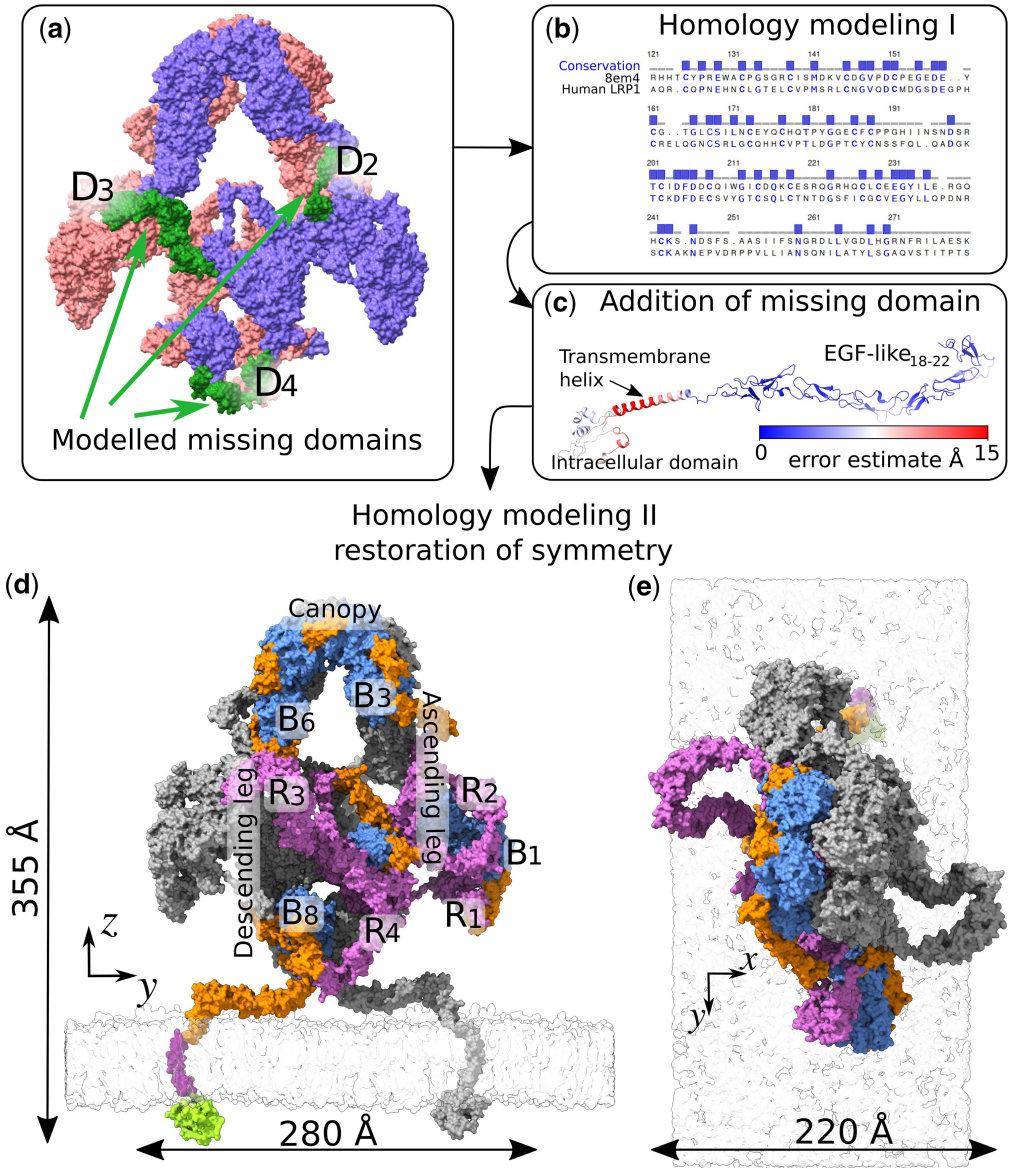
(a–c) Protocol steps for the dimeric structure prediction of LRP1. The steps are explained in the text. (d) Structure of the LRP1 dimer obtained by homology modeling from PDB 8EM4. The forefront monomer follows the representation in Fig. 1, while the background monomer is in grey. Bn and Rn indicate the β-propeller and the CB domains, respectively. (e) Dimer view from z-axis perspective.

Resolving the extensive inter-domain overlaps observed in the AlphaFold3 full-length model would require large rigid-body displacements of multiple domains, for which no experimental constraints are available. This said, we still notice a strong structural agreement between the full-protein AlphaFold3 local domains and the ones produced by RoseTTAFold, including the transmembrane and citoplasmatic domains (Fig. 4, available as supplementary data at *Bioinformatics* online). We therefore assembled the structure from independently predicted domains (Fig. 5, available as supplementary data at *Bioinformatics* online), preserving motif organization. Based on the reconstructed model, no stable contacts between extracellular domains and the transmembrane region are expected due to the spatial separation between the ectodomain and the membrane-proximal region. Calcium ions were positioned at conserved coordination sites identified from LDLR-family alignments (Fig. 1b), and disulfide bridges were added to maintain domain connectivity; these steps were followed by MD relaxation. The resulting model (Fig. 2a) displays a modular, flexible architecture that can extend to nearly 100 nm without altering local tertiary motifs. It is available in ModelArchive: https://www.modelarchive.org/doi/10.5452/ma-k8036. The 52 predicted N-glycans, shown in Fig. 2b, correspond to simplified high-mannose structures used for subsequent simulations.

### 3.3 The furin cleavage site lies within a stable β-propeller motif

LRP1 is processed by furin at the B8 motif, generating the extracellular α-chain and the transmembrane β-chain (Fig. 4a). We evaluated the strength of the non-covalent interactions holding the two chains together by isolating the B8 domain (Asp3779–Gln4235) and performing umbrella sampling simulations (see Section 2). The umbrella sampling extremes, in terms of reaction coordinate, are shown in Fig. 4b–d. The potential of mean force (PMF) revealed that dissociation requires approximately 180±2 kT (Fig. 4e), indicating that spontaneous chain separation under physiological conditions is unlikely. This estimate aligns with experimental evidence showing that soluble LRP1 (sLRP1) maintains its α–β association even under shear stress in circulation (Quinn *et al.* 1997, 1999). Thus, furin cleavage produces a heterodimer that remains stably linked through strong non-covalent interactions mediated by the β-propeller 8 motif.

### 3.4 Homology model of dimeric LRP1

**Figure 6:**
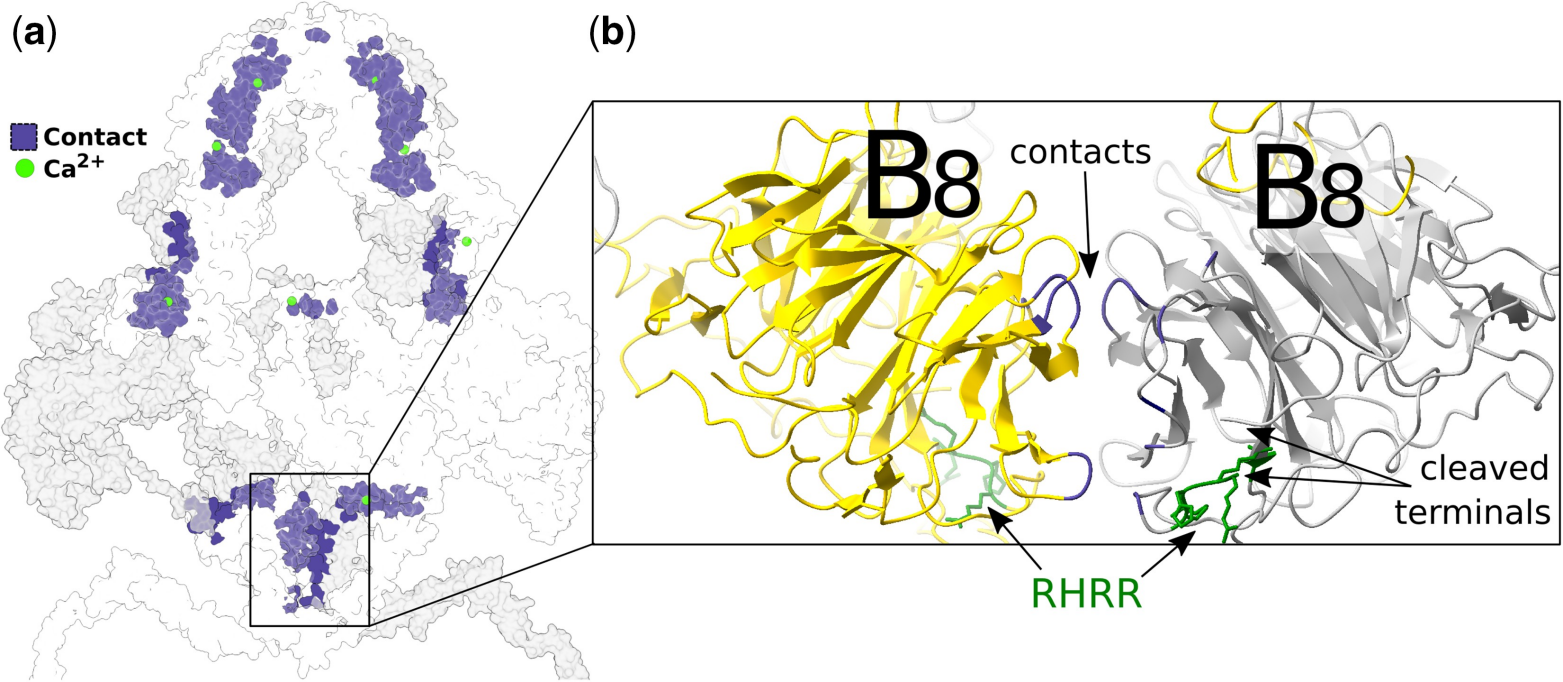
(a) Dimeric LRP1 shown as vdW transparent surface; with transparent surfaces allow the visualization of hidden contact points among the two monomers. (b) Zoom in on the lower intra-dimer interface between the two B8, in yellow and grey cartoon, respectively. In green, the two subsequences RHRR after furin cleavage. The interactive amino acids at the interface are colored in violet.

The dimeric model of LRP1 was generated by homology modeling using the mouse LRP2 cryo-EM structure (PDB 8EM4) as a template (Fig. 5a, Fig. 6, available as supplementary data at *Bioinformatics* online). Missing segments were reconstructed with RoseTTAFold and verified with AlphaFold2 predictions. Domains D2, D3, and D4 were connected to the corresponding positions in the template, while transmembrane and cytoplasmic domains were modelled de novo. The resulting structure is available in ModelArchive at https://www.modelarchive.org/doi/10.5452/ma-ubwf7. It retains the main architectural features of LRP2, a large quaternary assembly with two intertwined ectodomains connected at multiple contact points (Fig. 5d and e).

The interface area between protomers is 5951 **Å**^2^, corresponding to 1.23% of the total surface (Fig. 6a). The main interaction occurs within the canopy region, which forms a continuous lateral interface between β-propeller motifs (Fig. 6b). Additional contacts are observed between B8 motifs, suggesting a potential stabilizing role for this domain in the dimeric structure. As for the monomeric model, no stable contacts between extracellular domains and the transmembrane region are expected.

### 3.5 Flexibility and calcium coordination of the R domains

**Figure 7:**
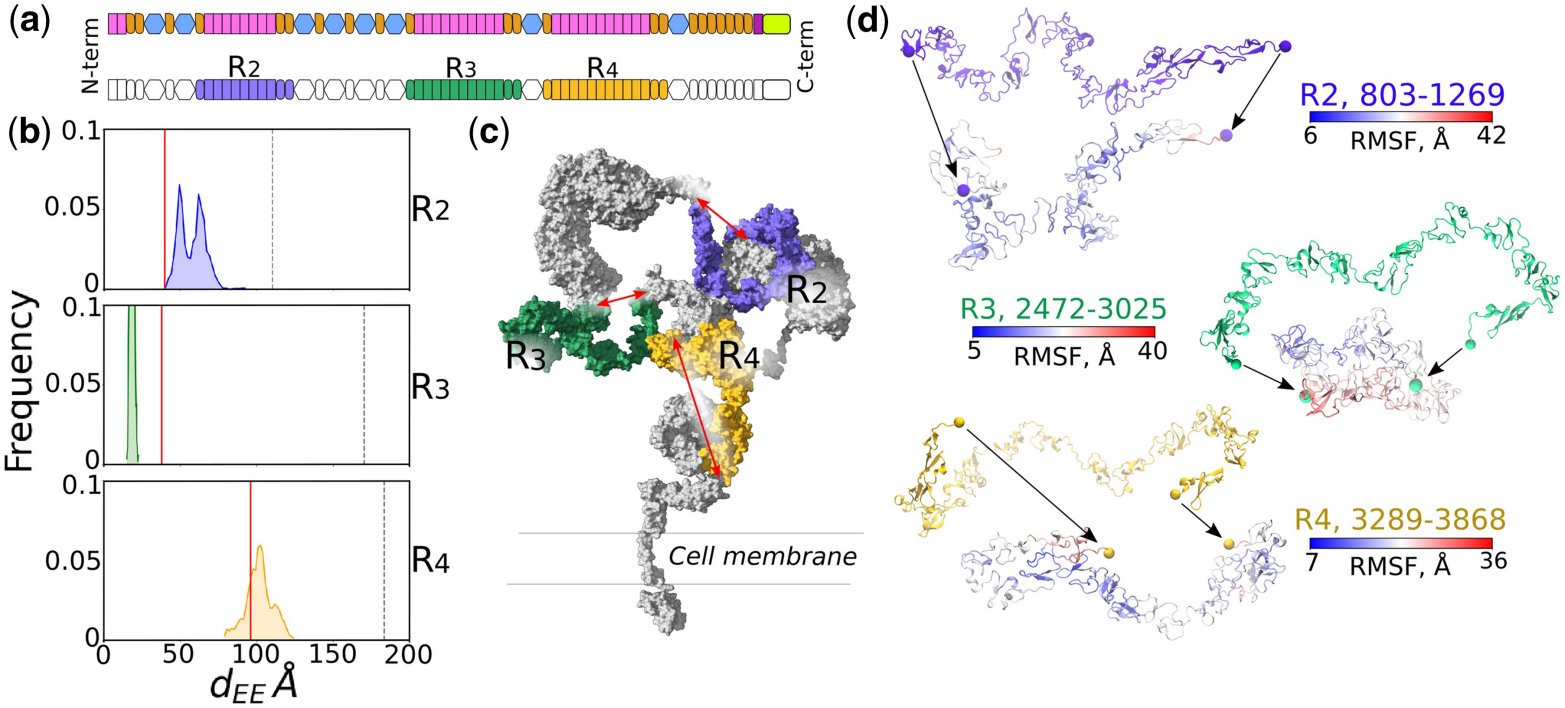
(a) Schemes of LRP1 motif sequence. Top: same scheme as Fig. 1. Bottom: Same scheme showing the macrodomains R2, R3, and R4. (b) Normalized frequency distribution of the Rn domain end-to-end distance dEE during the last 200 ns of MD simulations. The vertical dotted line represents the dEE value in the initial conformation; the red solid line is the dEE value for the R domain in the dimeric conformation. (c) Visual representation of LRP1 monomer in vdW surface. The three domains, R2, R3, and R4, define a static end-to-end distance, highlighted by the arrows. (d) Initial and final conformations of the R domains. The initial conformation is depicted using the color scheme; the final conformation is colored according to the calculated RMSF of each amino acid relative to the initial conformation. The arrows follow the translation of the domain terminals, depicted with vdW spheres.

**Figure 8:**
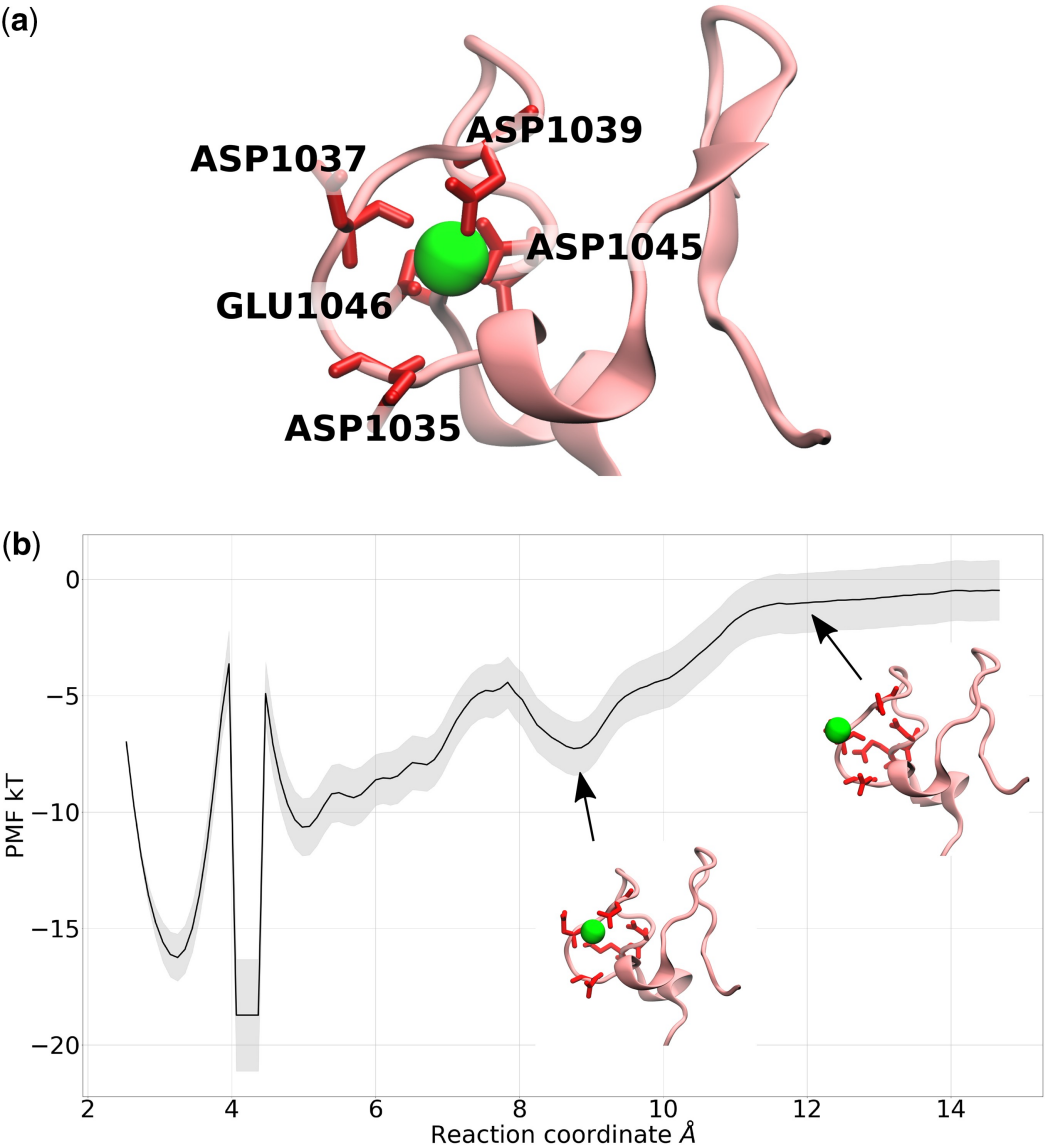
(a) CB 7 from R2 displayed as cartoon with key amino acids and calcium ionshighlighted. (b) Energy profile from the umbrella sampling protocol. The shaded area shows the uncertainty of the measure. The inset cartoons show local configurations of the ion.

We next analyzed the dynamic behavior of the flexible R2, R3, and R4 regions (Fig. 1). Each region, simulated independently, tended to coil spontaneously from an extended conformation, reaching equilibrium configurations with shorter end-to-end distances (d_EE_) (Fig. 7b). At equilibrium, d_EE_ values were comparable to those observed in the dimeric model (Fig. 7c), supporting the structural plausibility of the dimer arrangement. No significant differences were detected between glycosylated and non-glycosylated domains. During all simulations, Ca^2+^ ions remained bound to their coordination cages, confirming their high local stability. Umbrella sampling on the CB7 motif (Fig. 8a) yielded a PMF of 16±2 kT between the bound and unbound states (Fig. 8b), consistent with strong, persistent ion coordination.

### 3.6 Glycosylation stabilizes the dimeric canopy region

**Figure 9:**
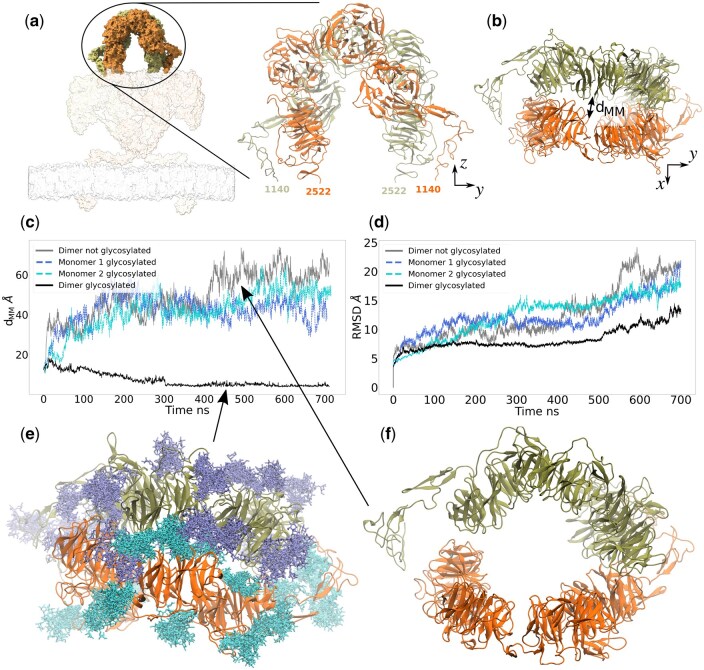
(a) Detail of the dimeric canopy structure (b) The xy view of the dimeric canopy highlights the protein vicinity, quantified by the distance dMM. (c) dMM as a function of time for the four systems: glycosylated dimer, asymmetrically glycosylated monomers, and non-glycosylated. (d) Root Mean Square Distance of heavy atoms for the four systems as a function of time. (e) Glycosylated canopy structure after 500 ns; with the N-glycans highlighted bonded to the two proteins. Ten conformations are shown for each glycan, highlighting the space occupied during the system evolution. The proteins keep their initial distance. (f) Non-glycosylated canopy structure after 500 ns. The proteins are mainly detached, creating a pore-like structure.

**Figure 10:**
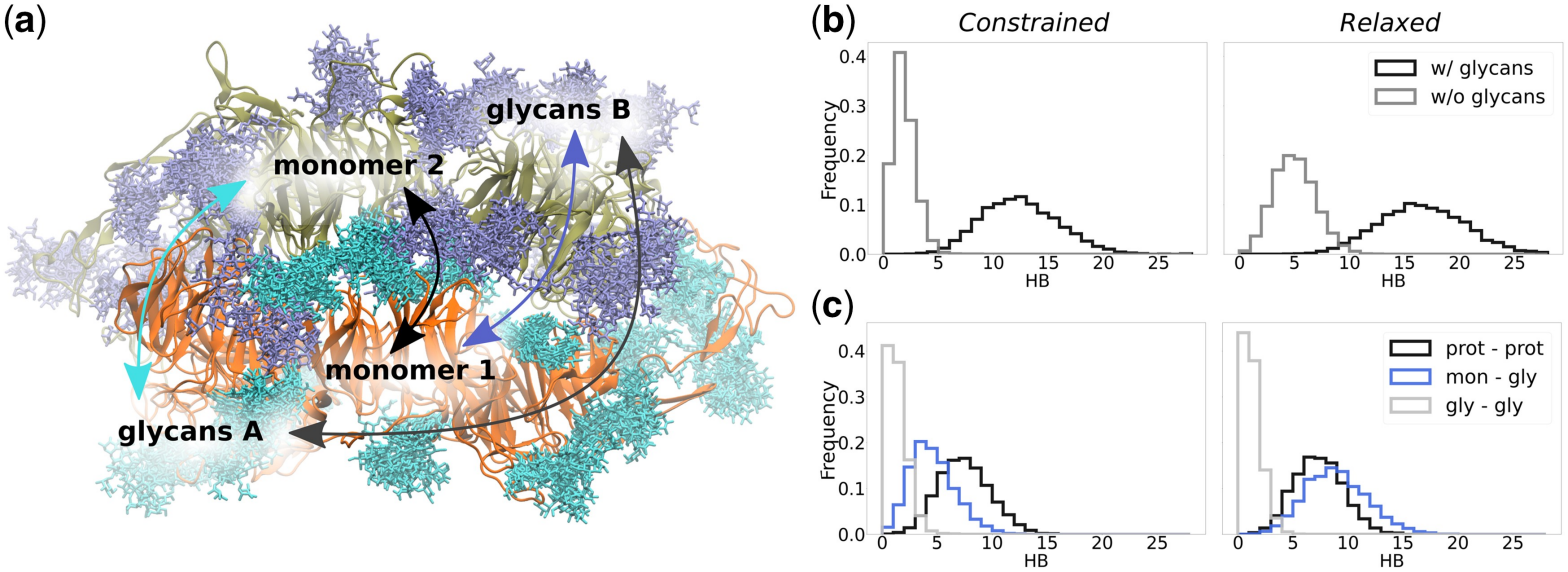
(a) Detail of the dimeric canopy structure in closed conformation at the end of the simulated time (700 ns). The monomers 1 and 2 are highlighted with the respective glycan groups, A and B, represented as licorice and anchored to monomer A and B, respectively. (b) The distribution of hydrogen bonds was calculated in the constrained and relaxed set-up for the closed and open conformations. In the closed conformation, fully glycosylated, the protein-protein, protein-glycan and glycan-glycan components were summed. In the open conformation, only the protein-protein HB was calculated. (c) The HB distribution for the three components in the fully glycosylated system: protein-protein, protein-glycan and glycan-glycan interactions. The second is the sum of monomer 1—glycan B and monomer 2—glycan A contributions.

The canopy region of LRP1 includes β-propellers B3–B6 and adjacent EGF-like motifs, forming the major inter-protomer interface (amino acids 1140–2522, Fig. 9a). To assess the effect of the 38 N-glycans on this interface (19 per monomer, see Table 2, available as supplementary data at *Bioinformatics* online), we simulated the canopy dimer in four configurations: fully glycosylated, asymmetric (single-monomer glycosylation), and non-glycosylated. Each system was evolved for 700 ns under identical conditions. Glycosylation markedly enhanced inter-protomer stability, maintaining a compact closed conformation, while unglycosylated systems adopted an open, more flexible state (Fig. 9e and f). Time-resolved monomer–monomer distances (d_MM_, Fig. 9b) and RMSD profiles (Fig. 9c and d) confirmed this distinction. Hydrogen-bond analyses revealed that fully glycosylated dimers formed an average of four additional hydrogen bonds relative to unglycosylated ones, primarily involving protein–glycan interactions (Fig. 10c). Glycan–glycan contacts were rare but contributed marginally to stability. In contrast, the open, non-glycosylated conformation displayed fewer and less persistent hydrogen bonds, particularly between β-propellers B3 and B6. Overall, glycans act as molecular stabilizers, reinforcing the closed quaternary arrangement through specific hydrogen bonding and steric constraints.

### 3.7 Sequence conservation supports the structural model

Evolutionary analysis using ConSurf (Glaser *et al.* 2003) revealed that residues buried within β-propeller cores are highly conserved, whereas surface residues show lower conservation except at interfacial regions (Fig. 7, available as supplementary data at *Bioinformatics* online). Residues located at the dimer interface are more conserved than other surface-exposed positions, suggesting evolutionary pressure to maintain inter-protomer contacts. Interestingly, conserved residues within the canopy region form fewer stable hydrogen bonds in non-glycosylated systems, whereas less conserved residues participate in stronger interactions when glycans are present (see Table 3, available as supplementary data at *Bioinformatics* online). This observation indicates that glycosylation complements, rather than replaces, the role of conserved amino acids in maintaining dimer stability.

## 4 Discussion

We present the novel hypothesis of a multi-quaternary structure for LRP1. The STORM analysis highlights the possibility for LRP1 to cluster on the surface of brain endothelial cells. With the available data, it is not possible to affirm what interaction exists between two or more LRP1 molecules, nor whether it is direct or mediated by other entities; however, the observation of groups of LRP1 indicates the presence of an interaction (Fig. 3). Based on these results, we produced two novel structural models for LRP1, describing both the monomeric and homodimeric conformations.

In the absence of significant experimental data on the tertiary structure, the monomeric structure of LRP1 is presented as a multi-domain flexible protein inserted into the cell membrane, consistent with LDLR family topology, although the relative orientation of domains remains uncertain due to their flexibility. Flexibility highly depends on the three calcium-binding domains R2, R3, and R4, which likely facilitate the receptor’s ability to adapt to multiple ligand-binding events. The monomeric organization and domain arrangement are shown in Fig. 2. MD simulations beyond initial energy minimization were restricted to selected extracellular domains, which were analyzed in isolation to enable adequate conformational sampling. During energy minimization, the transmembrane helix of the models was not explicitly embedded in a lipid bilayer. While membrane coupling may influence global orientation and long-timescale dynamics, it is not expected to significantly affect the local stability of calcium-binding domains or glycan-mediated interactions analyzed here. Full-length models were therefore not used for dynamical analysis but only for structural refinement and global architectural interpretation. The full-length models serve as structural scaffolds to contextualize the fragment-level simulations.

LRP1 is shed into two chains, α and β, by furin in the trans-Golgi compartment (Willnow *et al.* 1996). The shedding site appears in the β-propeller 8 (B8) motif.

The high stability of the B8-mediated α–β association (∼180±2 k_B_ T) supports previous observations of persistent chain coupling under physiological conditions (Fig. 4) (Quinn *et al.* 1997, 1999). Even in blood circulation, under the application of strong shear and torque forces, the non-covalent bond of the α and β chains is conserved in sLRP1.

The homodimer model was generated using a combination of neural-network-based structure prediction and homology modeling, employing LRP2 Cryo-TEM model as a homologous template. Based on this, the dimeric LRP1 model is consistent with biological evidence and represents a plausible arrangement rather than a purely physical construction. The resulting structure retains the same features as the template: a quaternary architecture, intra-dimer contacts between rigid domains, and more flexible regions exposed to solvent. We also propose plausible positions for the transmembrane and intracellular domains, which are absent in the LRP2 structure. Dimerization of LRP1 has not yet been observed experimentally; however, this unconfirmed dimerization raises the intriguing possibility that LRP1 could form heterodimers with other LDL receptor family members, thereby expanding its potential functional repertoire (Figs 5 and 6).

The two structural models enabled further dynamic characterization by MD simulations. Our first analysis studied the time evolution of the flexible domains R2, R3, and R4, isolated from the rest of the protein and artificially elongated while retaining all secondary structures. These domains tended to coil spontaneously, and the final conformations of R2, R3, and R4 show, at equilibrium, an end-to-end distance comparable to the same domains in the homodimer structure (Fig. 7). This observation supports the structural plausibility of the dimeric model. We did not observe changes in the coiling behavior influenced by the presence of glycans. We also observed calcium stability in the R domains’ coordination sites: not a single calcium ion left its cage during the MD simulations, indicating that Ca^2+^ ions provide strong local stabilization of domain geometry. Umbrella sampling analysis confirmed that the energy barrier for calcium release is approximately 16±2 k_B_ T, consistent with the observed persistence of bound ions (Fig. 8). These results suggest that the combination of local rigidity ensured by calcium and global flexibility of the connecting domains allows LRP1 to adapt conformationally to diverse ligands without structural destabilization.

We then analyzed the canopy region of the LRP1 homodimer, which comprises four β-propeller motifs per monomer arranged in a “U”-shape and interacting laterally through β-propeller contacts. Since this region is highly glycosylated, we tested the stability of the native interactions in the absence and presence of glycans. The presence of glycans notably increases the number of intra-dimer interactions, stabilizing the quaternary structure in a compact closed conformation. Without glycans, the hydrogen bonds between the two proteins are less stable, and the dimer adopts a more open configuration. These results indicate that glycans act as molecular stabilizers that strengthen inter-protomer contacts and modulate conformational equilibrium (Figs 9 and 10).

Evolutionary sequence analysis of the canopy β-propellers (Fig. 7, available as supplementary data at *Bioinformatics* online) revealed high conservation of residues compared with the rest of the protein, with core residues being more conserved than surface residues. Conserved residues correspond to known YWTD motifs (Jeon *et al.* 2001). Among canopy domains, β-propellers 3 and 6 display the highest conservation, suggesting an evolutionary bias in their structural roles. These conservation patterns further suggest that glycosylation compensates for divergent surface mutations, influencing the balance between open and closed dimer states. Dynamic analysis of intra-dimer hydrogen bonds showed that conserved residues form weaker and less persistent interactions in the absence of glycans, while less conserved residues participate in stronger hydrogen bonds when glycans are present. This indicates that glycosylation contributes more significantly to dimer stability than conserved canopy residues alone.

Together with our other observations, we conclude that glycans play a fundamental role in promoting dimerization and shaping the quaternary conformation of LRP1. Glycans act as a quaternary “glue” that stabilizes the dimeric interface, while calcium coordination preserves local folding integrity, enabling reversible clustering and high-avidity ligand binding at the cell surface. Such glycan-mediated stabilization may facilitate LRP1 clustering and modulate avidity-driven cargo recognition across the BBB. From an experimental perspective, the structural models presented here provide quantitative insight into the size and spatial organization of LRP1. The models, especially the homodimeric one, can serve as structural templates for the interpretation and refinement of low-resolution electron microscopy data (e.g. negative-stain or cryo-TEM), where the modular domain organization and overall shape can guide fitting procedures. The ectodomains span tens of nanometers, consistent with super-resolution imaging observations, and support the interpretation of receptor clustering at the cell surface. The identification of a canopy interface stabilized by glycan-mediated interactions suggests specific regions that could be targeted in mutagenesis or glycosylation-disruption experiments to test the proposed dimeric arrangement. Furthermore, the estimated free energy required to disrupt the α–β association (∼180 k_B_ T) provides a quantitative benchmark for receptor stability under mechanical or biochemical perturbations. These results therefore offer experimentally testable hypotheses linking receptor organization, glycosylation, and transport efficiency across the BBB.

## Supplementary Material

btag357_Supplementary_Data
