# Supplementary material for: A computational analysis of the glycoprotein lipoprotein receptor-related protein 1 structure and the role of glycans as quaternary glue
Source: Bioinformatics. 2026 Jun 4;42(7):btag357. doi: 10.1093/bioinformatics/btag357 (PMC13403279; doi:10.1093/bioinformatics/btag357)
Supplement: btag357_Supplementary_Data [file btag357_supplementary_data.pdf]

A computational analysis of the glycoprotein LRP1 structure and the role of glycans as quaternary glue.

Supporting Information

Immunofluorescence staining of bEnd.3 cells

Mouse brain endothelial cells (bEnd.3) were seeded onto fibronectin-coated μ-Slide 18-well glass-bottom chambers (IBIDI) and cultured to confluence for 3 days. Cells were rinsed with PBS and fixed with 3.75% (w/v) paraformaldehyde for 10 minutes at room temperature. To preserve surface-exposed LRP1, no permeabilisation was performed. Fixed cells were blocked with 5% bovine serum albumin (BSA) in PBS for 1 hour at room temperature and incubated overnight at 4 °C with mouse anti-LRP1 monoclonal antibody (Huabio, M1211-4) diluted 1:50 in 1% BSA in PBS. The following day, after three PBS washes, cells were incubated for 1 hour at room temperature with donkey anti-mouse IgG2a Alexa Fluor 647-conjugated secondary antibody (Abcam, ab150107) diluted 1:200 in 1% BSA in PBS. Nuclei were counterstained with Hoechst 33342 (1:1000 in PBS), followed by three final PBS washes. Samples were imaged immediately after staining.

STORM imaging set-up and secondary antibody calibration

To achieve stochastic optical reconstruction microscopy (STORM), samples were imaged in a GLOX buffer containing: (i) 2 μL of GLOX, (ii) 20 μL of MEA, and (iii) 20 μL of glucose (50% w/v) diluted in 160 μL of PBS (pH 7.4). This buffer provides an oxygen-scavenging environment suitable for 647-conjugated secondary antibodies, enhancing the blinking properties of Alexa Fluor 647 fluorophores.

To calibrate the secondary antibody and determine the number of localisations produced by a single fluorophore, goat anti-mouse IgG2a Alexa 647-conjugated secondary antibody was diluted 1:400,000 in PBS and deposited on a glass coverslip (Corning, thickness $1\frac{1}{2}$, 22 × 22 mm) for 10 minutes. After incubation, coverslips were washed several times with PBS to remove unbound antibody and imaged in the same GLOX buffer under the STORM conditions described below.

STORM imaging and Mean Shift Clustering analysis

STORM imaging of LRP1-labelled cells and antibody calibration samples was performed using a Nikon N-STORM system. Samples were illuminated with a 647 nm laser (160 mW), operated at 50% power for the first 10,000 frames and at 100% power for the subsequent 10,000 frames. Seven STORM movies were acquired for LRP1 samples and two for secondary antibody calibration. Fluorescence was collected with a Nikon 100× oil-immersion objective (numerical aperture 1.49), and emission was filtered through a quad-bandpass dichroic (Nikon 97335). Images were captured over a 256 × 256 pixel region (pixel size 0.16 μm) using a Hamamatsu ORCA Flash 4.0 camera with an integration time of 10 ms.

STORM movies were analysed in Nikon NIS Elements software using 2D Gaussian fitting. Localisation thresholds were set at 200 and 250, corresponding to photon count differences between signal peaks and background pixels. The trace length parameter was

set to 5 to prevent counting repeated blinking events from the same molecule: molecules visible in up to five consecutive frames were counted once, while longer events were excluded. Signals with more than five localisations were considered valid for molecular quantification.

Cluster identification was performed using Mean Shift Clustering (MSC) with radii of 50, 100, and 200 nm. Outliers were removed from the MSC results using the ROUT method (Q=1). Descriptive statistics of the cluster distributions, including mean, median, and percentile values, were computed to evaluate the number and distribution of localisations within LRP1 clusters.

Molecular Dynamics Setup and Parameters

Prior to molecular dynamics simulations, the assembled LRP1 models were subjected to a global energy minimization step to remove residual steric clashes and structural strain introduced during model construction. Minimization was performed using OpenMM (v8.0) [[Eastman et al., 2017]], employing the AMBER ff14SB force field [[Maier et al., 2015]] for the protein and GLYCAM06j-1 [[Kirschner et al., 2008] for glycans. All degrees of freedom were allowed to relax under the force-field potential. Implicit solvation was described using the GB-Neck2 model [[Nguyen et al., 2013]] with a non-bonded cutoff of 1 nm. The minimization was continued until convergence below 1 kJ mol^{-1}. The resulting relaxed structures were subsequently used for system preparation and molecular dynamics simulations in GROMACS.

All further MD simulations were performed using GROMACS 2021.2–2022.4 [[Abraham et al., 2015]] with the CHARMM36m force field [[Huang et al., 2017]]. Systems were solvated in a cubic box with explicit TIP3P water [[MacKerell et al., 1998]] and 0.15 M KCl to ensure electroneutrality. Each system was minimised using the steepest-descent algorithm (tolerance of 1000 kJ mol^{-1} nm^{-1}) with backbone and side-chain restraints of 400 and 40 kJ mol^{-1}, respectively. For glycosylated systems, 4 kJ mol^{-1} dihedral restraints were applied to glycan torsions.

Following minimisation, systems were equilibrated through multiple steps:

- **NVT equilibration:** 125 ps (for B8 systems) or 10 ns (for R-domain and canopy systems) with a 1 fs timestep using the velocity-rescale thermostat (time constant 1 ps, target temperature 303.15 K).
- **NPT equilibration:** 0.5–50 ns, with pressure maintained at 1 bar using the Parrinello–Rahman barostat (time constant 5 ps, compressibility 4.5×10^{-5} bar^{-1}).
- **Production:** 100–700 ns depending on the system, with a 2 fs timestep and LINCS-constrained bonds.

Non-bonded interactions employed a 1.2 nm cut-off for van der Waals forces with force-switching at 1.0 nm. Electrostatics were calculated with the Particle Mesh Ewald (PME) method (real-space cut-off 1.2 nm, grid spacing 0.12 nm). All simulations were carried out at 303.15 K and 1 bar.

System-specific Details

B8 domain simulations. The B8 domain (Asp3779–Gln4235) was simulated to quantify the energy required to separate the α and β chains. Pulling simulations were performed using a harmonic potential of 300 kJ mol^{-1} nm^{-2} for 25 ns at a rate of 10 nm ns^{-1}. The ensemble was NPT, with Parrinello–Rahman pressure coupling (0.5 ps) and Nose–Hoover temperature coupling (1 ps). Twenty-six umbrella windows spaced by 0.2 nm were simulated for 10 ns each, followed by WHAM reconstruction of the potential

of mean force (PMF). Statistical uncertainty was assessed via Bayesian bootstrap with 200 resamplings [[Hub et al., 2010]].

Flexible domain simulations (R2–R4). Three flexible regions were simulated independently:

- R2: residues 803–1269 (CB3–CB10, EGF-like4–6)
- R3: residues 2472–3025 (CB11–CB20, EGF-like10–12)
- R4: residues 3289–3868 (CB21–CB31, EGF-like13–15)

Each domain was equilibrated for 60 ns (10 ns NVT + 50 ns NPT) before 100 ns of NVT production. The timestep was 1 fs during equilibration and 2 fs during production. No terminal constraints were applied. RMSF and end-to-end distance (d_{EE}) distributions were calculated using MDAnalysis [[Michaud-Agrawal et al., 2011]] and visualised in VMD [[Humphrey et al., 1996]].

Calcium extraction from CB7. The CB7 motif was extracted from the R2 cluster (residues 1030–1050). After equilibration, a harmonic pulling potential of 1000 kJ mol^{-1} nm^{-2} was applied for 2 ns to dissociate the Ca^{2+} ion. Twenty-five umbrella windows spaced by 0.2 nm were simulated for 10 ns each. The PMF between bound and unbound states was obtained using WHAM [[Kumar et al., 1992]], with statistical error from 200 bootstrap resamples.

Canopy region simulations. Residues 1140–2522 of each monomer (covering β-propellers B3–B6 and EGF-like5–10) were simulated as a dimer in four glycosylation states:

1. Fully glycosylated (19 sites per monomer)
2. Asymmetric glycosylation of monomer A only
3. Asymmetric glycosylation of monomer B only
4. Non-glycosylated

Glycans ($GlcNAc_2Man_5$) were built using Glycosylator [[Lemmin and Soto, 2019]] and manually adjusted to remove steric clashes. Each simulation consisted of 500 ns constrained production followed by 200 ns unrestrained evolution. Analysis of monomer–monomer distance (d_{MM}), RMSD, and hydrogen-bond counts was performed using MDAnalysis [[Michaud-Agrawal et al., 2011]].

Evolutionary Conservation

Residue conservation was computed using ConSurf [[Glaser et al., 2003]]. A multiple sequence alignment of 150 LRP1 orthologues was built from UniProt using MAFFT. Conservation scores were normalised and mapped onto the canopy β-propeller structures using PyMOL. Highly conserved residues (scores ≥ 8) were compared with interfacial hydrogen bonds observed in MD trajectories to assess evolutionary constraints on dimer stability.

Table S1. Summary of all molecular dynamics simulations performed in this study.

System	Residues	Length (ns)	Glycosylation State
B8 (chain separation)	3779–4235	26 × 10	None
R2	803–1269	160	None
R3	2472–3025	160	None
R4	3289–3868	160	None
CB7 (Ca^{2+} extraction)	1030–1050	25 × 10	None
Canopy (fully glycosylated)	1140–2522 × 2	700	$GlcNAc_2Man_5$
Canopy (asymmetric A)	1140–2522 × 2	700	Partial
Canopy (asymmetric B)	1140–2522 × 2	700	Partial
Canopy (non-glycosylated)	1140–2522 × 2	700	None

Simulation Summary

Software and Visualization

Visualisations were generated with Chimera [[Pettersen et al., 2021]], PyMOL [[Schrödinger and DeLano,]], and VMD [[Humphrey et al., 1996]]. Plots were produced using Matplotlib and NumPy in Python 3.10. All input files, parameters, and starting coordinates are available upon reasonable request.

Figures

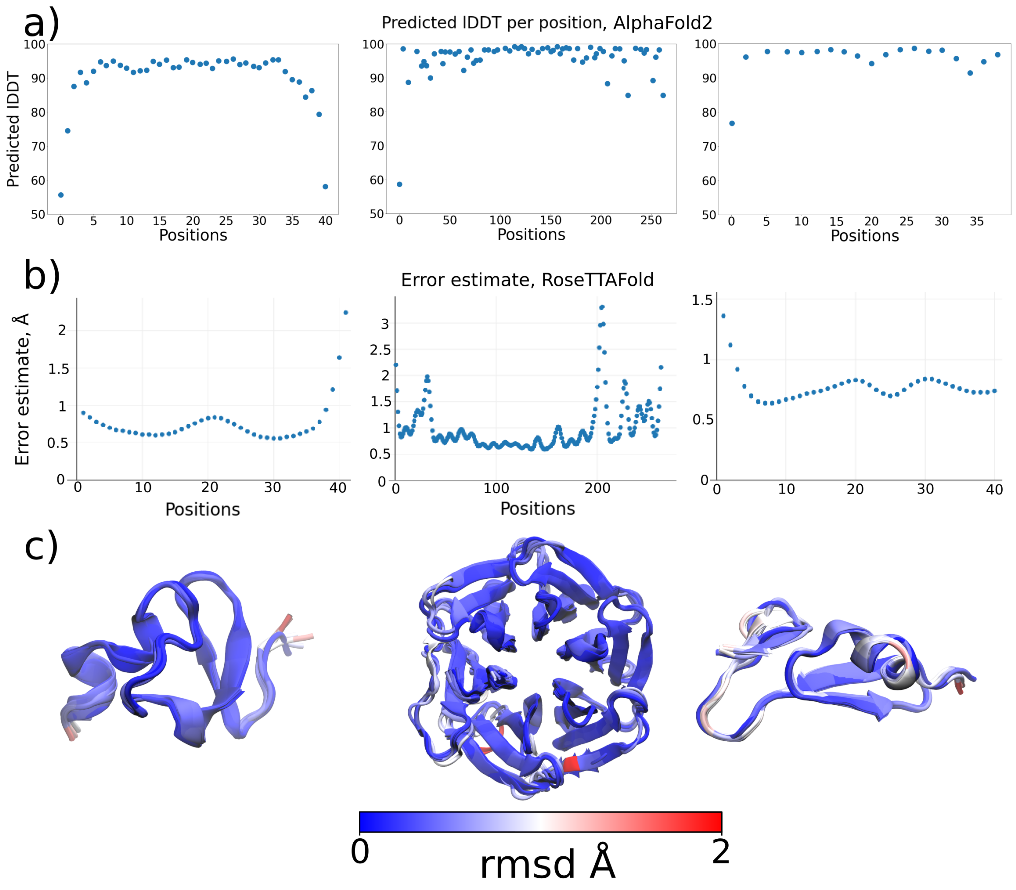

Figure S1. Evaluation of LRP1's predicted motifs using structure predicting algorithms. a) Predicted local distance difference test LDDT per position of the AlphaFold2 prediction for each motif, from left to right: calcium-binding motif, β-propeller motif, EGF-like motif. Values above 90 correspond to very high confidence of the structure prediction. b) Error estimate from the RoseTTAFold prediction for each motif, following the order in (a). c) Structural alignment of the five RoseTTAFold and five AlphaFold2's prediction of the three motifs, following the order in (a). The amino acids are highly super-imposed, demonstrating the two neural network's prediction quality.

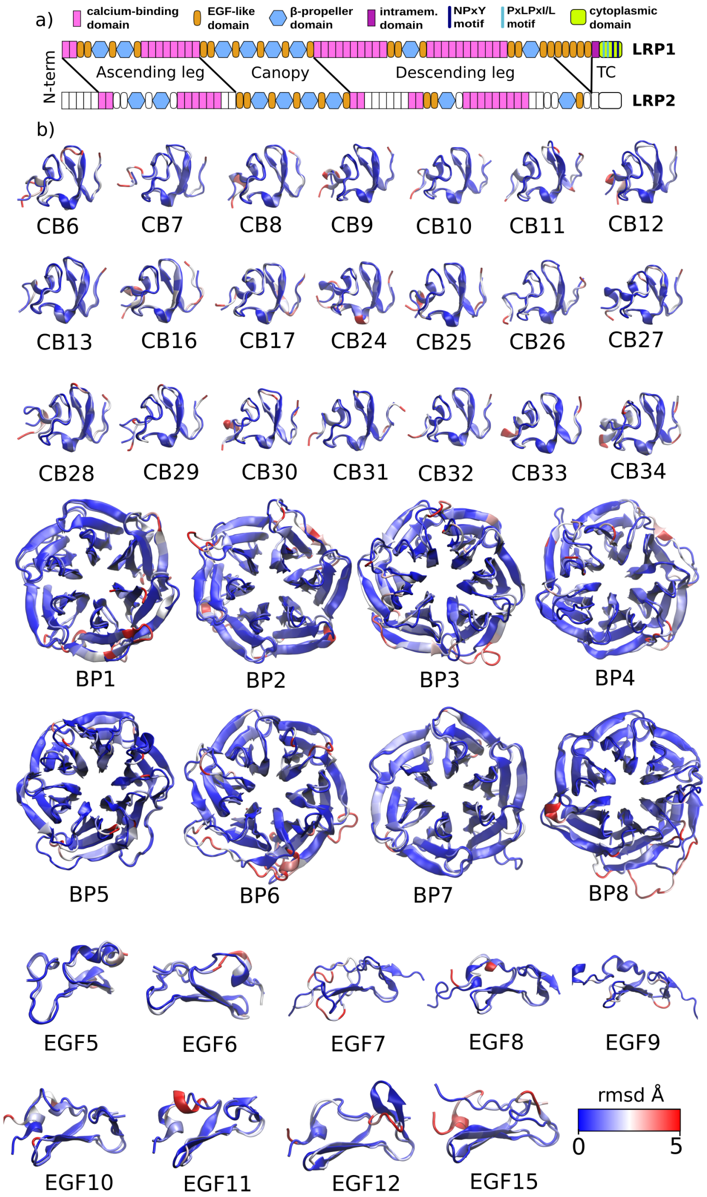

Figure S2. Evaluation of LRP1's predicted motifs using structure predicting algorithms. a) Alignment of the LRP1 and LRP2 structural sequences. It is evident the similarity between the two motif sequences, if ignoring the first five CB units in LRP2 (counting from N-terminal) and the last five EGF-like motifs in LRP1. The motifs in white in LRP2 are the ones not used by 8EM4 for the analysis. b) Structural superimposition of the motifs from the cryo-TEM observation 8EM4 and the corresponding motif in the LRP1 neural network prediction (RoseTTAFold). Each couple satisfyingly superimpose, confirming the validity of the NN in the local prediction.

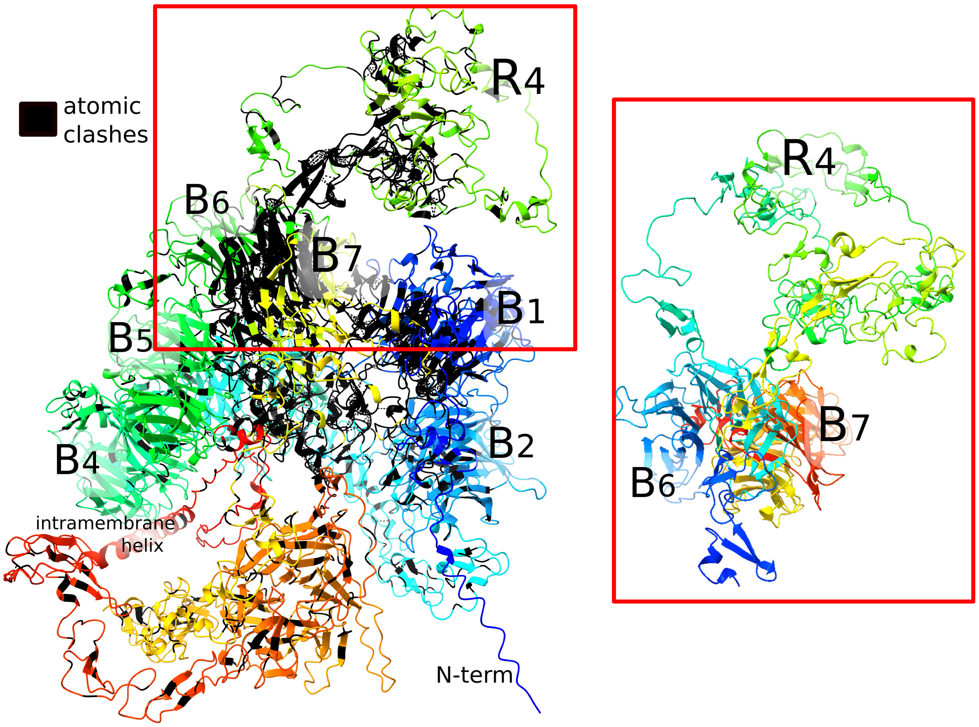

Figure S3. Evaluation of AlphaFold3 full LRP1 prediction. Prediction of the LRP1 full protein by AlphaFold3, shown as a cartoon and colored with rainbow colors from N terminus (blue) to C terminus (red). The N terminus and the intramembrane helix close to the C terminus are visible. LRP1 has a coiled conformation with many contacts (amino acids in black) and even a superimposition of the β-propellers 6 and 7, highlighted by the red square.

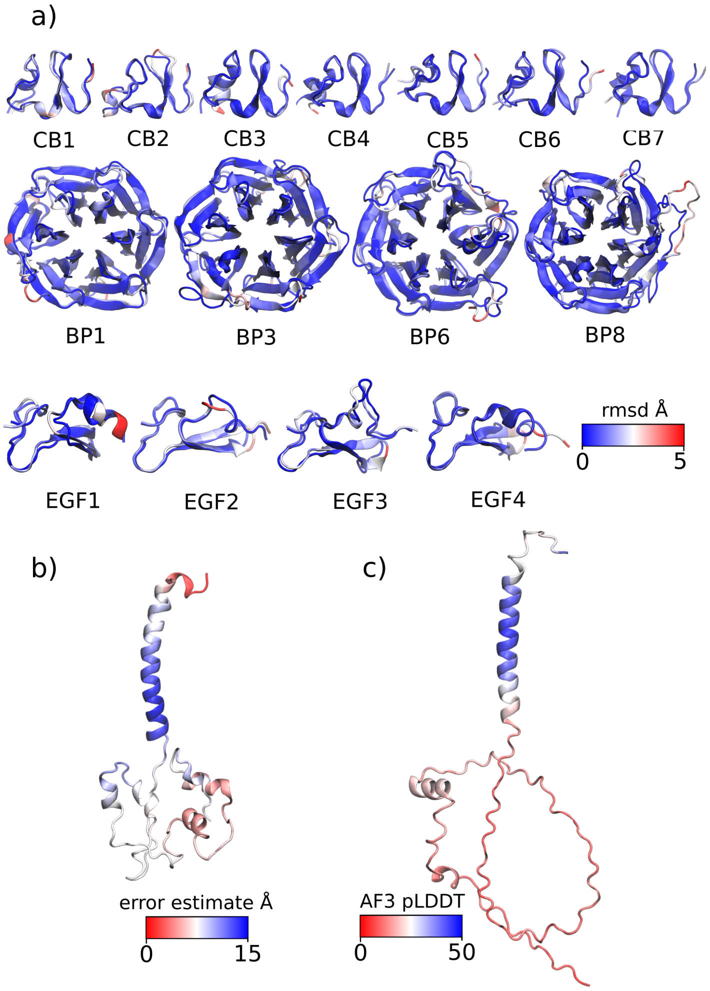

Figure S4. Evaluation of AlphaFold3 full LRP1 prediction with respect to the monomeric LRP1 obtained in this work. (a) Superimposition of representative calcium-binding (CB), β-propeller (BP), and EGF-like motifs predicted independently using RoseTTAFold (blue) and AlphaFold3 (colored by C-α RMSD, blue–red scale). The high structural agreement at the local level indicates that both methods reliably predict folded domains despite differences in global organization. (b) Transmembrane and intracellular domains of LRP1 predicted independently using RoseTTAFold, showing a structured and membrane-compatible conformation. Color represents the error estimate from RoseTTAFold prediction. (c) The same regions extracted from the full-length AlphaFold3 prediction. Color represents AlphaFold3 pLDDT (predicted local distance difference test).

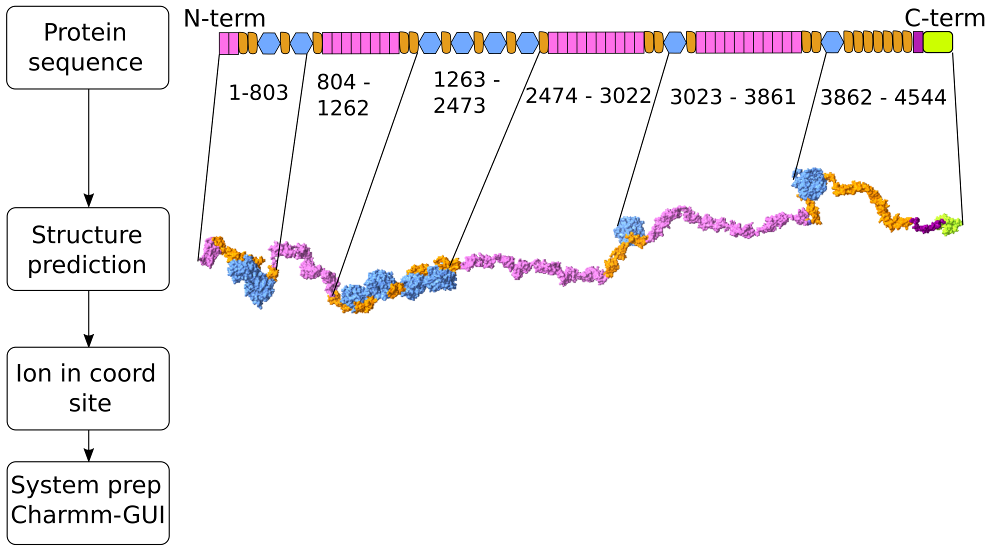

Figure S5. Production of a single LRP1 by joining of protein fragments. Protocol for the production of the LRP1 monomeric model. We first separated the protein sequence into six portions and produced for each a predicted structure using RoseTTAFold. The fragments were then joined and adjusted in the conformation shown here and in Fig. 3.b. We further added calcium ions to the coordination sites of the calcium-binding motifs and, finally, prepared the glycoprotein for molecular dynamics simulations using the CHARMM-GUI web server [[Jo et al., 2008]].

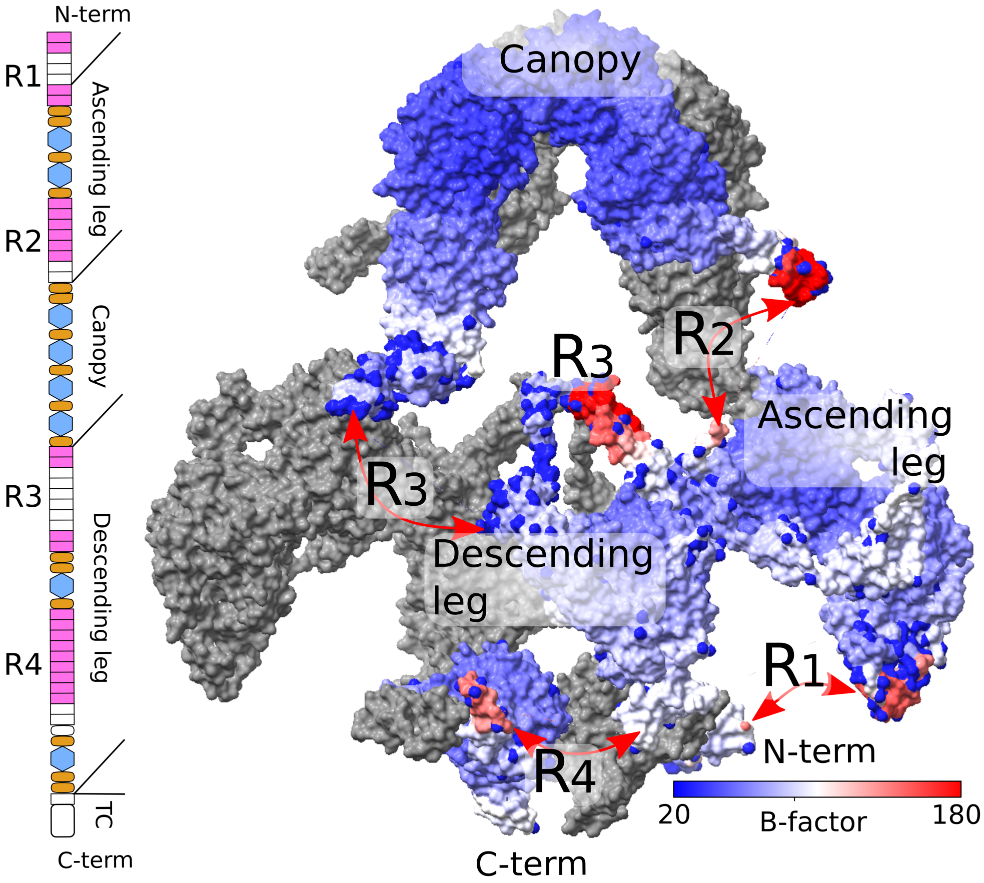

Figure S6. LRP2 cryo-TEM structure B-factor enhances the flexibility of specific domains. LRP2 at pH 7.5 (PDB 8EM4) obtained by the observations of Beenken et al [[Beenken et al., 2023]]. The two proteins of the dimer are shown in vdW surface; the forefront monomer displays the atomic B-factor values, and the background is colored grey. The red indicates a greater variability of the atomic positions. Notably, the atoms in calcium-binding domains R1, R2, R3 and R4 are more prone to move; some portions of the domains are missing because the atomic positions were not defined with enough high resolution, highlighting an overall greater flexibility of the R domains compared with the rest of the protein. The white units of the scheme on the left show the missing units of 8EM4.

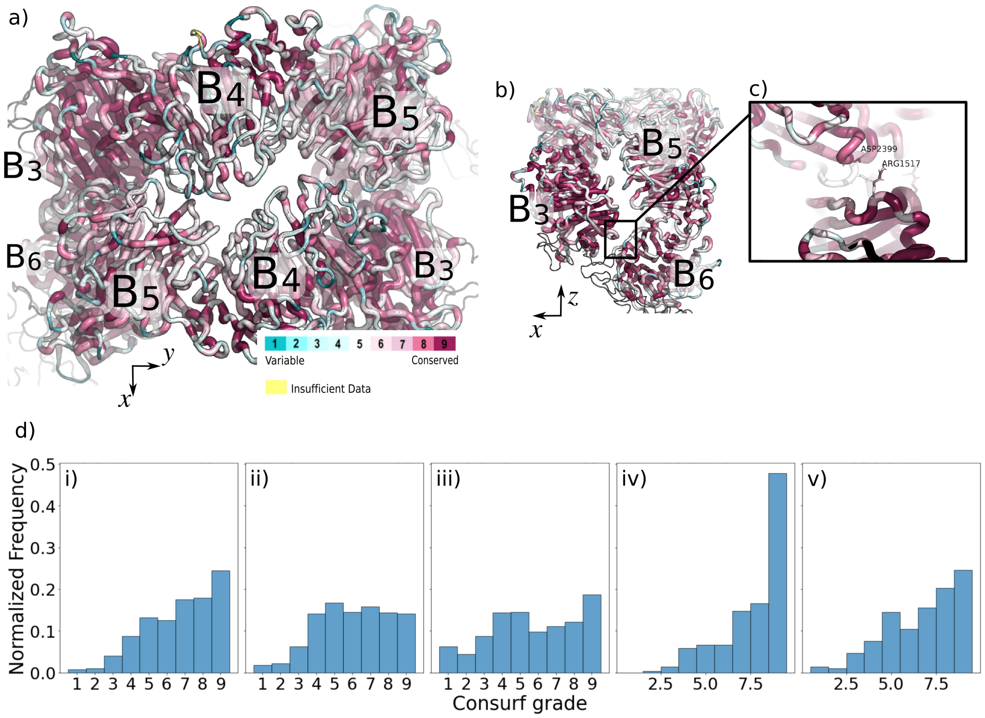

Figure S7. Evolutionary conservation. a) Top view of the LRP1 dimeric canopy. Amino acids are colored following the ConSurf [[Glaser et al., 2003]] scheme (See Methods). High scores indicate the residue is highly conserved among identified homologue protein structures, indicating functional importance. A general trend is observed that core residues of the β-propellers are more conserved than the ones at the surface. Lower scores of surface residues indicate their non-functional role at the dimeric interface. b) The most conserved amino acids at the interface are between β-propellers 3 and 6. c) Detail of amino acids ASP2399 and ARG1517, the most conserved among the ones involved in hydrogen bonds (see Tab. S3). d) Histograms showing the probability density of finding a Consurf grade: i) in the canopy β-propellers (3 to 6); ii) in the β-propellers outside the canopy (1, 2, 7, 8); iii) in the full protein, excluding the canopy; iv) in β-propeller 3; v) in β-propeller 6.

Tables

LRP1 N-glycan sites	114, 136, 185, 239, 274, 357, 446, 729, 928, 1050, 1154, 1155, 1195, 1218, 1511, 1558, 1575, 1616, 1645, 1723, 1733, 1763, 1825, 1933, 1995, 2048, 2117, 2127, 2472, 2502, 2521, 2539, 2601, 2620, 2638, 2815, 2905, 3048, 3089, 3264, 3333, 3488, 3662, 3788, 3839, 3953, 4075, 4125, 4179, 4278, 4279, 4364

Table S2. N-glycosylation sites (Asparagines) in LRP1. In red, the glycans belonging to the canopy region used in MD simulations.

Glycosylated			Glycosylated - relaxed		
HB	Occ %	ConSurf score	HB	Occ %	ConSurf score
ARG1497 GLU2418	93.2	8-5	ARG1966 ASP1407	87.6	7-9
ARG1517 GLU2418	87.9	7-5	TYR2030 PRO1411	61.5	3-9
TYR2030 PRO1411	61.8	3-9	TYR2030 SER1409	54.9	3-6
TYR2030 SER1409	57.1	3-6	LYS1788 GLU1899	53.3	4-6
**Not glycosylated**			**Not glycosylated - relaxed**		
HB	Occ %	ConSurf score	HB	Occ %	ConSurf score
ARG1517 GLU2418	39.5	7-5	ARG1517 GLU2418	59.1	7-5
ARG1517 ASP2399	21.2	7-9	ARG1517 ASP2399	41.6	7-9
LYS2454 GLU1476	19.6	6-8	LYS2416 PHE1477	30.2	5-5
ARG1517 PRO2419	15.2	7-8	ARG1454 ASP2008	29.9	7-8

Table S3. First four intra-dimer hydrogen bonds, ordered by stability, for the glycosylated and non-glycosylated canopy, before (400-500ns) and after (600-700ns) the relaxation of terminal constraints. For each amino acid couple, we listed the ConSurf score.

References

Abraham et al., 2015. Abraham, M. J., Murtola, T., Schulz, R., Páll, S., Smith, J. C., Hess, B., and Lindah, E. (2015). Gromacs: High performance molecular simulations through multi-level parallelism from laptops to supercomputers. *SoftwareX*, 1-2:19–25.

Beenken et al., 2023. Beenken, A., Cerutti, G., Brasch, J., Guo, Y., Sheng, Z., Erdjument-Bromage, H., Aziz, Z., Robbins-Juarez, S. Y., Chavez, E. Y., Ahlsen, G., Katsamba, P. S., Neubert, T. A., Fitzpatrick, A. W., Barasch, J., and Shapiro, L. (2023). Structures of lrp2 reveal a molecular machine for endocytosis. *Cell*, 186:821.

Eastman et al., 2017. Eastman, P., Swails, J., Chodera, J. D., McGibbon, R. T., Zhao, Y., Beauchamp, K. A., Wang, L. P., Simmonett, A. C., Harrigan, M. P., Stern, C. D., Wiewiora, R. P., Brooks, B. R., and Pande, V. S. (2017). Openmm 7: Rapid development of high performance algorithms for molecular dynamics. *PLOS Computational Biology*, 13:e1005659.

Glaser et al., 2003. Glaser, F., Pupko, T., Paz, I., Bell, R. E., Bechor-Shental, D., Martz, E., and Ben-Tal, N. (2003). Consurf: identification of functional regions in proteins by surface-mapping of phylogenetic information. *Bioinformatics (Oxford, England)*, 19:163–164.

Huang et al., 2017. Huang, J., Rauscher, S., Nawrocki, G., Ran, T., Feig, M., Groot, B. L. D., Grubmüller, H., and MacKerell, A. D. (2017). Charmm36m: An improved force field for folded and intrinsically disordered proteins. *Nature methods*, 14:71.

Hub et al., 2010. Hub, J. S., Groot, B. L. D., and Spoel, D. V. D. (2010). G-whams-a free weighted histogram analysis implementation including robust error and autocorrelation estimates. *Journal of Chemical Theory and Computation*, 6:3713–3720.

Humphrey et al., 1996. Humphrey, W., Dalke, A., and Schulten, K. (1996). Vmd: Visual molecular dynamics. *Journal of Molecular Graphics*, 14:33–38.

Jo et al., 2008. Jo, S., Kim, T., Iyer, V. G., and Im, W. (2008). Charmm-gui: A web-based graphical user interface for charmm. *Journal of Computational Chemistry*, 29:1859–1865.

Kirschner et al., 2008. Kirschner, K. N., Yongye, A. B., Tschampel, S. M., González-Outeiriño, J., Daniels, C. R., Foley, B. L., and Woods, R. J. (2008). Glycam06: A generalizable biomolecular force field. carbohydrates. *Journal of computational chemistry*, 29:622.

Kumar et al., 1992. Kumar, S., Rosenberg, J. M., Bouzida, D., Swendsen, R. H., and Kollman, P. A. (1992). The weighted histogram analysis method for free-energy calculations on biomolecules. i. the method. *Journal of Computational Chemistry*, 13:1011–1021.

Lemmin and Soto, 2019. Lemmin, T. and Soto, C. (2019). Glycosylator: A python framework for the rapid modeling of glycans. *BMC Bioinformatics*, 20:1–7.

MacKerell et al., 1998. MacKerell, A. D., Bashford, D., Bellott, M., Dunbrack, R. L., Evanseck, J. D., Field, M. J., Fischer, S., Gao, J., Guo, H., Ha, S., Joseph-McCarthy, D., Kuchnir, L., Kuczera, K., Lau, F. T., Mattos, C., Michnick, S., Ngo, T., Nguyen, D. T., Prodhom, B., Reiher, W. E., Roux, B., Schlenkrich, M., Smith, J. C., Stote, R., Straub, J., Watanabe, M., Wiórkiewicz-Kuczera, J., Yin, D., and Karplus, M. (1998). All-atom empirical potential for molecular modeling and dynamics studies of proteins. *Journal of Physical Chemistry B*, 102:3586–3616.

Maier et al., 2015. Maier, J. A., Martinez, C., Kasavajhala, K., Wickstrom, L., Hauser, K. E., and Simmerling, C. (2015). ff14sb: Improving the accuracy of protein side chain and backbone parameters from ff99sb. *Journal of Chemical Theory and Computation*, 11:3696–3713.

Michaud-Agrawal et al., 2011. Michaud-Agrawal, N., Denning, E. J., Woolf, T. B., and Beckstein, O. (2011). Mdanalysis: A toolkit for the analysis of molecular dynamics simulations. *Journal of Computational Chemistry*, 32:2319–2327.

Nguyen et al., 2013. Nguyen, H., Roe, D. R., and Simmerling, C. (2013). Improved generalized born solvent model parameters for protein simulations. *Journal of chemical theory and computation*, 9:2020–2034.

Pettersen et al., 2021. Pettersen, E. F., Goddard, T. D., Huang, C. C., Meng, E. C., Couch, G. S., Croll, T. I., Morris, J. H., and Ferrin, T. E. (2021). Ucsf chimerax: Structure visualization for researchers, educators, and developers. *Protein science : a publication of the Protein Society*, 30:70–82.

Schrödinger and DeLano, . Schrödinger, L. and DeLano, W. Pymol.
